# Cannulation in extracorporeal membrane oxygenation

**DOI:** 10.1186/cc13959

**Published:** 2014-07-01

**Authors:** Orhan Gokalp, Yuksel Besir, Bortecin Eygi, Gamze Gokalp, Levent Yilik, Ali Gurbuz

**Affiliations:** 1 Department of Cardiovascular Surgery, Faculty of Medicine, Izmir Katip Celebi University, Altinvadi Cd. No. 85, D:10, Narlidere, Izmir, 35360, Turkey; 2 Department of Cardiovascular Surgery, Ataturk Education and Research Hospital, Karabaglar, Izmir, 35360, Turkey; 3 Department of Pediatric Emergency Medicine, Izmir Tepecik Education and Research Hospital, Tepecik, Izmir, 35370, Turkey

## 

We sincerely appreciate the studies performed about mobilization in extracorporeal membrane oxygenation (ECMO) patients [1]. An increasing number of studies can be seen recently in this very challenging group of patients. Mobilizing an ECMO patient is a complicated task to due to surrounding equipment and the nature of cannulations [2,3]. Regarding postoperative mobilization, the most comfortable side for cannulation is the subclavian artery/femoral vein, as indicated by Abrams and colleagues [1]. There is no significant contraindication to mobilize patients with femoral arterial/venous cannulation but the procedure should be slightly difficult, as Abrams and colleagues stated. The question here is whether we should place the cannulae considering the possibility of postoperative mobilization. Does this possibility really have to affect our choice of cannulation side?

ECMO is a life-saving tool although it comes with many different complications, including cannulation-related bleeding, ischemia of ipsilateral extremity, vascular injury and infection [4]. Can the cannulation side make a difference and reduce the number of complications?

Abrams and colleagues suggest subclavian artery cannulation for easier mobilization, although this also sometimes comes with unexpected complications. We would like to report a patient treated with ECMO in our clinic who had some complications never reported previously in the literature. ECMO was established through the subclavian artery with a flow of 3,500 ml/minute but the patient had cerebral and pupil edema, so the flow was decreased to 2,000 ml/minute and the right arm was elevated – the symptoms regressed spontaneously. The patient was weaned from ECMO 8 days later and decannulated.

Cannulation sides may have advantages over one another although this does not suggest that we should go for a routine subclavian cannulation as indicated by Abrams and colleagues. We would like to hear opinions and experiences about similar complications regarding subclavian cannulation.

## Authors’ response

Darryl Abrams, Matthew Bacchetta and Daniel Brodie

We thank Gokalp and colleagues for raising the issue of the optimal cannulation configuration for patients requiring venoarterial ECMO in whom mobilization is anticipated, and for highlighting potential issues with subclavian arterial reinfusion [1]. Although mobilization may be attempted with femoral cannulation, the only data regarding the safety of such mobilization have been reported with smaller central venous catheters rather than those used for ECMO [5]. For that reason, our preferred approach for ambulatory venoarterial ECMO, with few exceptions, is the combination of internal jugular venous drainage and subclavian arterial reinfusion via an end-to-side graft [6,7]. The surgical technique needs to be meticulously executed to withstand patient mobilization and to function for days to weeks. The anastomotic approach differs from that which is commonly employed in the operating room for cardiothoracic surgeries [8]. In addition, when choosing the initial extracorporeal blood flow, it is important to consider the physiologic needs of the patient. High levels of venoarterial blood flow early on may have adverse consequences, including compromise of graft integrity and impairment of left ventricular emptying due to excessive afterload. Effective use of extracorporeal life support requires both well-executed cannulation and patient-focused management of the device. Ultimately, the choice of configuration has to balance the overall goals of therapy with the potential risks of that particular cannulation strategy.

## Abbreviation

ECMO: Extracorporeal membrane oxygenation.

## Competing interests

DB reports receiving research support from Maquet Cardiovascular including travel expenses for research meetings and anticipated support for upcoming studies and compensation paid to Columbia University for research consulting; he receives no direct compensation from Maquet. DB is also a member of the Medical Advisory Board for ALung Technologies and compensation is paid to Columbia University; he receives no direct compensation from ALung Technologies. MB reports receiving research support from Maquet Cardiovascular including travel expenses for research meetings and anticipated support for upcoming studies and compensation paid to Columbia University for research consulting; he receives no direct compensation from Maquet. The remaining authors declare that they have no competing interests.
